# Stereotactic, single-dose irradiation of stage I non-small cell lung cancer and lung metastases

**DOI:** 10.1186/1748-717X-1-30

**Published:** 2006-08-20

**Authors:** Peter Fritz, Hans-Jörg Kraus, Werner Mühlnickel, Udo Hammer, Wolfram Dölken, Walburga Engel-Riedel, Assad Chemaissani, Erich Stoelben

**Affiliations:** 1 St. Marien-Krankenhaus, (Medical Education Hospital of the University of Marburg), Department of Radiotherapy, D-57072 Siegen, Germany; 2 St. Marien-Krankenhaus, (Medical Education Hospital of the University of Marburg), Department of Radiology, D-57072 Siegen, Germany; 3 Cologne Metropolitan General Hospital, Clinic for Thoracic Disease, D-51109 Köln, Germany

## Abstract

**Background:**

We prospectively reviewed response rates, local control, and side effects after non-fractionated stereotactic high single-dose body radiation therapy for lung tumors.

**Methods:**

Fifty-eight patients underwent radiosurgery involving single-dose irradiation. With 25 patients, 31 metastases in the lungs were irradiated; with each of 33 patients, stage I non-small cell lung cancer (NSCLC) was subject to irradiation. The standard dose prescribed to the isocenter was 30 Gy with an axial safety margin of 10 mm and a longitudinal safety margin of 15 mm. The planning target volume (PTV) was defined using three CT scans with reference to the phases of respiration so that the movement span of the clinical target volume (CTV) was enclosed.

**Results:**

The volume of the metastases (CTV) varied from 2.8 to 55.8 cm^3 ^(median: 6.0 cm^3^) and the PTV varied from 12.2 to 184.0 cm^3 ^(median: 45.0 cm^3^). The metastases ranged from 0.7 to 4.5 cm in largest diameter. The volume of the bronchial carcinomas varied from 4.2 to 125.4 cm^3^(median: 17.5 cm^3^) and the PTV from 15.6 to 387.3 cm^3 ^(median: 99.8 cm^3^). The bronchial carcinomas ranged from 1.7 to 10 cm in largest diameter. Follow-up periods varied from 6.8 to 63 months (median: 22 months for metastases and 18 months for NSCLC). Local control was achieved with 94% of NSCLC and 87% of metastases. No serious symptomatic side effects were observed. According to the Kaplan-Meier method the overall survival probability rates of patients with lung metastases were as follows: 1 year: 97%, 2 years: 73%, 3 years: 42%, 4 years: 42%, 5 years: 42% (median survival: 26 months); of those with NSCLC: 1 year: 83%, 2 years: 63%, 3 years: 53%, 4 years: 39%: (median survival: 20.4 months).

**Conclusion:**

Non-fractionated single-dose irradiation of metastases in the lungs or of small, peripheral bronchial carcinomas is an effective and safe form of local treatment and might become a viable alternative to invasive techniques.

## Background

Clinical practice since the 1980s and numerous publications have documented the indications and methodology involved in and the results obtained from cranial stereotactic irradiation techniques. The earliest reports on radiation treatment of extracranial targets involving stereotactic positioning are from Lax et al. [1] and Blomgren et al. [2], who describe a "stereotactic body frame for exact tumor localization and reproducible fixation", a frame developed at the Karolinska Hospital in Stockholm. In 1995 and in 1998, this Swedish research group published the first clinical results of hypofractionated stereotactic radiation treatment with high single doses. Major indications were primary tumors of the liver and of the lungs and metastases in the liver and lungs [1,2]. At the same time, laser-induced thermotherapy (LITT) or high frequency-induced thermotherapy (HITT) became attractive methods for local ablation of liver and lung tumors [3,4]. Findings of the first studies of LITT and HITT treatments of lung tumors have recently been published [5,6].

This paper reports on outcomes in 58 patients with metastases in the lungs or with histologically confirmed, small, peripheral, non-small-cell bronchial carcinomas, patients who underwent stereotactic single-dose body radiation therapy. At the date set for evaluation, all patients had undergone follow-up observation for at least 6.0 months.

## Methods

### Eligibility

Eligible patients had no more than two targets and sufficient pulmonary function (FEV 1 ≥ 1.0 l/s). Further criteria had to be fulfilled: Karnofsky performance status ≥ 60%, no proximity to high-risk organs, no signs of metastases in other organs, primary tumor under control (in cases of lung metastases). Histological confirmation was also required. Patients with metastases from all primary tumors were also included, with the exception of those from SCLC or germ cell carcinomas. Additional criteria for local radiation treatment of non-small-cell bronchial carcinomas (NSCLC) were as follows: peripheral site, no signs of local lymph node metastases or of remote metastases.

Radiation exposure to high-risk organs (trachea, esophagus, great vessels, spinal cord and skin) had to be < 10 Gy. At most, the planning target volume (PTV) (80% isodose) could only be allowed to affect the wall of a hollow organ along the length of the radiation field, but not any parts of the lumen. Since a fibrosis of the skin was to be avoided as far as possible, the distribution of the dose in the chest wall was determined so that a maximum of 30% of the isodose was allowed in the subcutis, i.e., 5 mm under the skin. If these specifications could not be put into practice with a corresponding treatment plan, the patient was offered stereotactic hypofractionated treatment (not described in detail in this paper). The proportion of patients who were originally scheduled for single-dose radiation therapy, but underwent hypofractionated radiotherapy instead was 22%.

For all of the patients, there were either severe health conditions or technical factors prohibiting conventional surgery or chemotherapy, or the patients had refused surgery or further attempts at chemotherapy. Stereotactic body radiation therapy was approved by the regional ethics committee and was in accordance with the Helsinki Declaration, as revised in 1983. All patients were informed about the experimental approach of this clinical trial and gave their written consent.

### Pretreatment assessment

All of the patients underwent a spirometric test (FEV 1), CT scans of the thorax and abdomen, total body bone scintigraphy, and laboratory examinations of blood count and serum enzymes. Since differential diagnoses of the coin-shaped density in the lungs can also include numerous benign results from pneumonia to granulomas, histological confirmation of malignancy was required. All occurrences of NSCLC and 55% of metastases were histologically confirmed by bronchoscopy or CT-guided trans-thoracic biopsy. All of the bronchial carcinomas and metastases scheduled for stereotactic single-dose irradiation were also subjected to flourodeoxyglucose positron emission tomography (PET). For NSCLC, PET was used primarily as a supportive, auxiliary examination method to rule out local hilar or mediastinal metastases. Yet, a hypermetabolism discovered in the PET was evaluated as a lymph-node metastasis only if the CT scan showed topographically correlated results. Classification of a coin-shaped density in the lungs as a metastasis, then, depended on the following criteria: known primary tumor, manifestation and growth observed in earlier CT scans, malign hypermetabolism exhibited in the PET scan, or histological confirmation related to the primary tumor.

### Defining the target volume and the radiation treatment regimen

**Figure 1:**
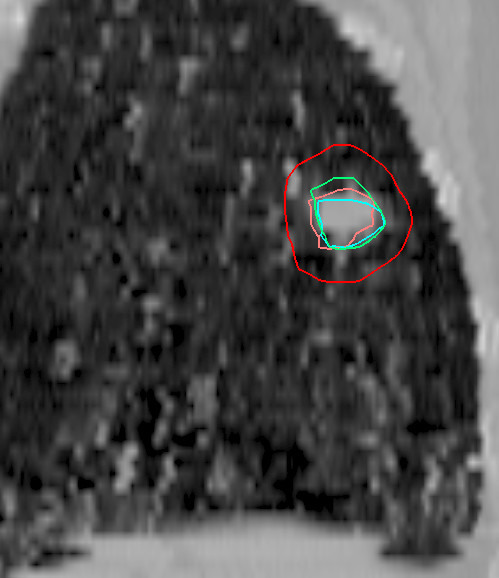
Image fusion of three dynamic CT scans in a reconstructed sagittal plane for determining target position (CTV) during respiration. (Green line: normal inhalation, pink line: naturally contracted position of the chest, blue line: normal exhalation, red line: PTV.) Tilting the tumor in x and y axes during respiration facilitates the phase-specific display of the CTVs.

Before beginning with stereotactic single-dose radiation treatment, the patient was positioned in the stereotactic body frame using the abdominal compression device. At first, a preliminary fluoroscopy was carried out. But even with the use of the abdominal compression device, most of the tumors exhibited noticeable respiratory motion. A treatment-planning CT scan of the thorax with 3 mm slice thickness was performed and displayed during 3 respiratory phases: normal inhalation, normal exhalation, and the naturally contracted position of the chest. These series of CT scans were then used to make an image fusion using the Eclipse 3-D planning system (version 7.3.10; Varian). The identical position of the patients and the classification of the slices through the localization markers in the body frame facilitated a first-rate image fusion of the CT scans. The clinical target volume (CTV) was determined by the macroscopically visible gross tumor volume (GTV) with the use of pulmonary windowing. For each set of CT scans, the CTV was marked in separately. The superimposition of the various tumor positions, depending on the respiratory phase, resulted in a presentation of the span of the tumor's movement. The definitive planning target volume (PTV) was then marked in around this movement span with a safety margin of 15 mm in the longitudinal plane and 10 mm in the transverse plane so that all of the active respiratory phases of the CTV were enclosed (Fig. 1).

The pencil beam algorithm of the Eclipse system was used for dose calculation and for 3-D treatment planning. The inhomogeneity correction was computed with the modified Batho power law, as implemented in Eclipse. The dose was prescribed to the isocenter. Five to eight coplanar, conformal fields were planned and were then irradiated, using a linear accelerator (Precise SLi, Elekta) with a multileaf collimator (leaf width: 10 mm).

**Figure 2:**
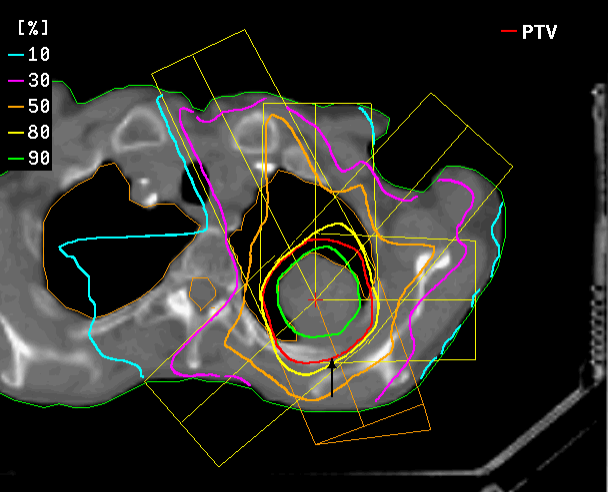
planning target volume (red line ) and dose distribution for single-dose irradiation of a bronchial carcinoma using six coplanar portals (course of remission see figures figures 3a, b, c, d).

The standard dose prescribed to the isocenter was 30 Gy. The definition of the target volume ensured that, of the prescribed isocenter dose, at least 90% covered the gross tumor volume (GTV = CTV) and at least 80% the PTV (Fig. 2). Irradiation was performed 3 to 4 days after the CT scan for treatment planning. Prior to irradiation, a second series of CT scans was performed in order to ensure the reproducibility of the patient's position and the position of the previously planned isocenter over the entire target. The slices displaying the isocenter and the target were compared with the CT treatment plan. All prominent anatomical structures were used for this comparison (pulmonary vessels, airways, bony structures, and the tumor image). Additionally, the patient's position in x and y directions, i.e., with reference to the bottom panel and to the side panels of the stereotactic frame, was measured using a distance cursor and compared to the specifications from the planning CT scan in order to rule out or correct changes in the patient's vertical and lateral position in the vacuum pillow. After this procedure, the patient was transported in the body frame to the linear accelerator. Here, a third check on the isocenter was carried out by comparing portal images (Iview system, Elekta) with virtually simulated images. Compared to the treatment plan, in all cases corrections of the isocenter coordinates in the longitudinal or transverse plane were less than 5 mm.

### Follow-up

All of the patients were checked using high-resolution helical CT scans of the entire lung at 6 and 12 weeks after the single-dose radiation treatment. Standard slice thickness was 8 mm to minimize the radiation exposure of the entire thorax, as there were frequent follow-up CT examinations. If suspect alterations occurred, additional CT scans with 3 mm slices were made so that a comparison with the high-resolution treatment-planning CT as a reference CT for follow-up was provided. Further follow-up examinations then took place in three-month intervals. In accordance with WHO criteria, tumor response was defined as complete if all abnormalities that were anatomically related to the tumor disappeared after treatment, and defined as partial if the maximum size of these abnormalities decreased by ≥ 50%. CT images were independently interpreted by three diagnostic radiologists. CT characteristics were determined on the basis of a consensus among at least two of the three radiologists. Respiratory functions (FEV 1) were measured in six-month intervals.

### Statistics

Statistical endpoints were overall survival and freedom from local failure according to the Kaplan-Meier method. Medcalc version 8.2.1.0, Schoonjans, was used for statistical analysis. Additionally, the initial response six months after radiotherapy was measured by volumetric analysis based on CT slices using the planning system Eclipse 7.3.10 (Varian).

## Results

### Patient collective

The follow-up period was defined as the time between irradiation and the last follow-up examination including a thoracic CT scan. The median follow-up period for patients with lung metastases was 22 months (periods extending from 6.8 to 63 months) and for patients with NSCLC 18 months (extending from 7.7 to 53.4 months). The median age for patients with lung metastases was 65 years (range: 32 – 82 years) and 72 years for patients with NSCLC (range: 59 – 82 years). Forty men and 18 women were treated. With reference to the origin of the metastases, data were as follows: 9 targets were metastases from rectal cancer, non-small cell lung carcinoma (n = 10), breast cancer (n = 6), ENT (n = 3), other origin (n = 3). Biopsies of bronchial carcinomas exhibited the following histological results: squamous cell carcinoma n = 15, adenocarcinoma n = 11, large cell carcinoma n = 5, NOS (not otherwise specified) n = 2. For all of the patients, there was no significant temporal relationship to previous chemotherapy. None of the patients underwent concurrent chemotherapy.

### Target volumes

The volume of the metastases (CTV) varied from 2.8 to 55.8 cm^3 ^(median: 6.0) and the PTV varied from 12.2 to 184.0 cm^3 ^(median: 45.0). The metastases ranged from 0.7 to 4.5 cm in largest diameter (median: 2.5 cm). In 6 patients, 2 lung sites were irradiated concurrently. The volume of the bronchial carcinomas varied from 4.2 to 125.4 cm^3 ^(median: 17.5) and the PTV from 15.6 to 387.3 cm^3 ^(median: 99.8). The bronchial carcinomas ranged from 1.7 to 10 cm in largest diameter (median: 5.5 cm).

### Control rates and survival

**Figure 3:**
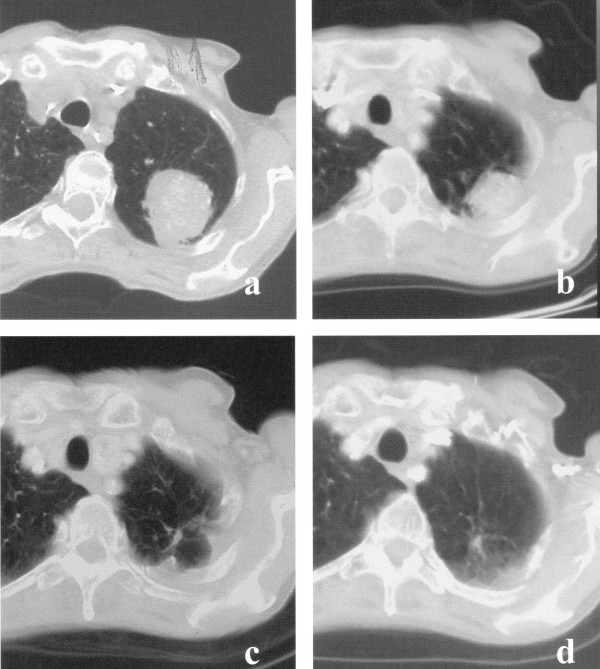
a, b, c, d: (a) bronchial carcinoma before single-dose irradiation with 30 Gy, (b) partial remission after 10 months, (c) complete remission 21 months after irradiation leaving a scarlike fibrosis. (d) dense consolidation after 48 months.

Since the regression of tumors, especially of larger ones, sometimes proceeds at a slow rate, the degree of initial remission was assessed 6 months after single-dose radiation treatment had occurred. Fifty-eight of sixty-four (90.6%) tumors responded to therapy (Fig. 3a,b,c,d). The extent of tumor response was difficult to measure in some cases due to the appearance of radiation-induced interstitial changes. The majority of these postradiation changes began at the site of the 30–40% isodose area. So in most cases, residual tumors could be distinguished from characteristics of radiation pneumonitis. On the other hand, we considered any suspect residual irregular density to be evidence of residual tumor tissue (partial remission). The actual complete remission rate, then, may be higher. In 17 out of 33 (51.5 %) patients with NSCLC, solid tumor formations completely disappeared and were classified as complete remission (CR). Fourteen patients (42.4 %) exhibited a partial remission (PR) and two NSCLC relapsed (6.0 %). Twenty out of 31 metastases showed a CR (64.5 %), 7/31 a PR (22.6%) and four metastases relapsed (12.9%) during further follow-up. In further follow-up after high-dose radiosurgery, distinguishing between tumor recurrence and radiation pneumonitis and fibrosis proved to be difficult.

Tumor progression or recurrence was assumed if a progressive increase of solidification within the planning target volume (PTV) could be observed between successive CT scans during a follow-up period of six months. The patients involved also had a PET scan performed. If the scan showed a hypermetabolism typical of tumors, a further biopsy or salvage operation was carried out. This situation occurred in five patients after radiotherapy (two cases of NSCLC and three of lung metastases), and, in all cases, the radiological and nuclear-medicine diagnosis was confirmed histopathologically.

**Figure 4:**
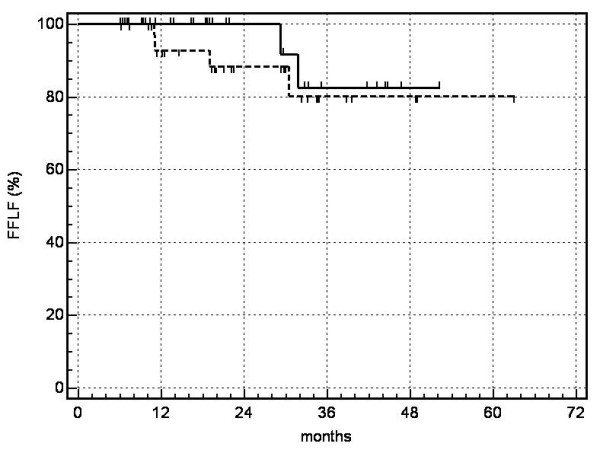
probability of local control (FFLF = freedom from local failure):---------- metastases,  NSCLC

**Figure 5:**
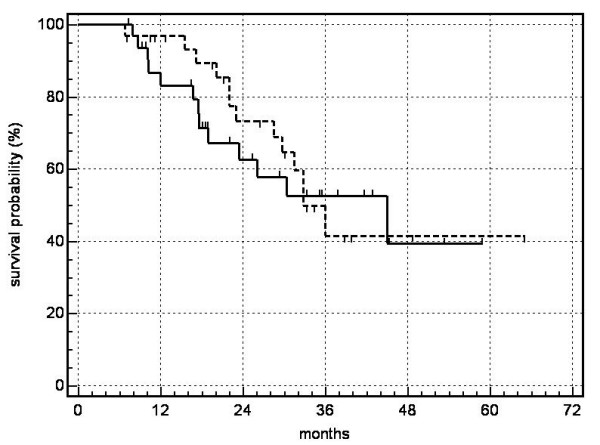
probability of overall survival : --------- metastases,  NSCLC.

On the date set for evaluation (March 31, 2006) or at the time of death, 21 patients with irradiated lung metastases and 24 patients with NSCLC had undergone a follow-up period > 12 months. Thirty-one of 33 NSCLC (94%) and 27 of 31 metastases (87%) manifested local control. Relative to the number of lesions irradiated, the probability of local control reached 80% at 5 years for metastases and 83% at 4 years for NSCLC (Fig. 4). The probability of overall survival for patients with metastases calculated according to Kaplan-Meier was as follows: one year: 97%, 2 years: 73%, 3 years: 42%, 4 years: 42%, 5 years: 42% (median survival 26 months); for patients with NSCLC: one year: 83%, 2 years: 63%, 3 years: 53%, 4 years: 39% (median survival 20.4 months) (Fig. 5). The most significant cause of death for the patients who died was systemic progression with occurrences of new metastases. At present, no significant difference in terms of local control and overall survival has been detected in comparing patients after irradiation of lung metastases or of primary lung cancer (log rank test).

### Side effects

**Figure 6:**
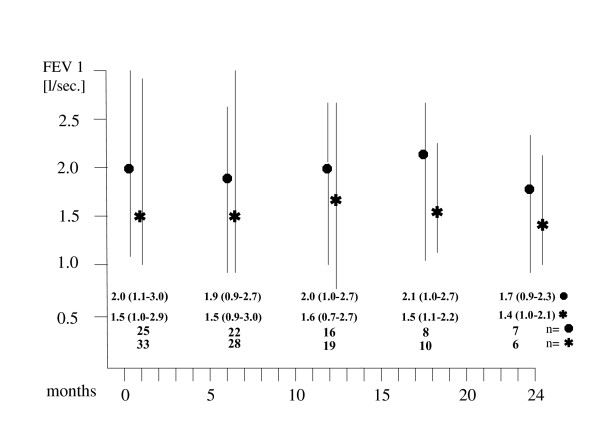
Respiratory functions (FEV 1) after stereotactic single-dose irradiation (●metastases, bronchial carcinomas, median and range values for patients (n) alive and available at the specified follow-up periods).

The median volume encompassed by the 9 Gy isodose was 9.1% of total lung volume (range: 2.2 to 23.1%). The 12 Gy and 15 Gy isodose encompassed 7.0% (range: 1.6 to 16.4 %) and 5.3% (range: 1.2 to 14.3%), respectively. Yet, these volumes and the planning target volumes were still small in comparison to the total volume of the lungs and, up to this point, have resulted neither subjectively nor objectively in a diminution of respiratory functions. No patient died due to respiratory insufficiency (Fig. 6).

Except for four cases of acute grade 1 radiation dermatitis (WHO- Toxicity Criteria) following radiation treatment of tumors situated near the thoracic wall, there were no symptomatic side effects. In particular, no patient had to be treated because of pneumonitis. At the time of response evaluation (six months after irradiation), 73% of our patients exhibited characteristics of radiation pneumonitis on the CT scans.

In 8 of 33 patients with NSCLC (24 %), CT scans revealed pneumonitic alterations in sites near the thorax wall associated with asymptomatic, cytologically benign, temporary pleural effusions, which, in contrast to malignant pleural effusions, were only of slight volume and disappeared after several months without treatment.

## Discussion

A number of studies on stereotactic body radiation therapy of lung tumors have been published. Several research teams that carried out hypofractionated, stereotactic irradiation, applied 3 to 10 fractions with doses per fraction varying from 6 to 20 Gy [7-10,12]. The cumulative referential doses of the hypofractionated series totaled 24 to 60 Gy. Moreover, doses were specified in widely divergent ways: prescribed to 65% isodose volume [2,11], to 80% isodose [10,12,13], or to the isocenter [9].

Some of the patients of Nakagawa et al. [8] and of Uematsu et al. [13] received conventionally fractionated radiation treatment, so that the 'stereotactic irradiation' only functioned as a 'boost'. It is difficult to compare all of these courses of treatment. Yet, the fractionated series all involve higher single doses irradiated under precise stereotactic conditions. The consistency of the results with high local control rates of 80–100% and with a very slight incidence of symptomatic side effects is especially noteworthy.

Non-fractionated single-dose irradiation is more convenient for the patient but the literature on the clinical experience of this approach is scanty. Results of single-dose irradiation of lung tumors have been published by Hara et al. [14] and Hof et al. [15] – studies involving patient collectives of 19 and 10 patients and mean follow-up periods of 13 and 14.9 months, respectively. Hof et al. [15] irradiated 10 histologically confirmed non-small-cell bronchial carcinomas < 5 cm with doses prescribed to the isocenter ranging from 19 to 26 Gy. Under respiratory gating, Hara et al. [14] irradiated 23 lung metastases or bronchial carcinomas < 4 cm with doses of 20 to 30 Gy specified to the edge of the tumor (GTV = CTV). Single-dose irradiation was also tested by Wulf et al. [16] as an alternative to hypofractionated sterotactic body radiation therapy for primary lung cancer and pulmonary metastases. Twenty-five metastases (and one NSCLC) were irradiated with 26 Gy prescribed to the 80%-isodose comprising the PTV, which corresponds to approximately 31.5 Gy at the isocenter (PTV = CTV plus 5 mm margins). During the follow-up (median: 9 months), no local failure was observed. Thus, our dosage was in accordance with the magnitude of reference doses reported by these authors. Our results correspond to those of Hara et al., Hof et al., and Wulf et al., who were also able to attain high local control rates ranging from 80 to 100%, with no serious symptomatic side effects. The post-radiation treatment changes in lung parenchymas shown in CT scans seem to exhibit a decelerated dynamics, different from that of the changes following conventionally fractionated radiation treatment. Unlike changes associated with conventional radiation therapy, the pulmonary opacities did not precisely correspond to the planned target volume, were often located at the periphery of the dose distribution, and could undergo change in shape and location for months. No pleural effusions were found after single-dose radiation treatment of lung metastases, which is probably related to the much smaller volumes of irradiation.

Two NSCLC and four metastases relapsed. The CTV of the metastases were 2.8 cm^3^, 6.5 cm^3^, 12.6 cm^3 ^and 32 cm^3^. Three of them were metastases from a rectal carcinoma (adenocarcinoma) and one from an adenoidcystic carcinoma. The CTV for the NSCLC that relapsed were 55 cm^3^(large cell carcinoma) and 32 cm^3 ^(squamous cell carcinoma). At present, as the number of relapses is small, we cannot see any patterns of recurrences in terms of target histology and CTV.

Introducing respiratory gating indicates problems of accuracy with respect to targets involving respiratory motion. Also, the time expenditure needed for respiratory gating can, in itself, prove to be an obstacle: Hara et al. specify this as one hour. Other authors used the abdominal compression device of the stereotactic body frame. Wulf et al. [17] investigated the "impact of target reproducibility on tumor dose in stereotactic radiotherapy of targets in the lung and liver" and found that "pulmonary targets with increased breathing mobility are at risk for target deviation exceeding the standard security margins for PTV-definition and require individual evaluation of sufficient margins." Hof et al. [18] concluded: "In our experience, the abdominal compression device integrated into the stereotactic frame limits diaphragmatic movement only inadequately." Our observations of widely divergent individual respiratory motion despite using the abdominal compression device correspond to these studies and confirm our original concept of defining the target volume with reference to the phases of respiration. Precisely for single-dose irradiation, 100% target coverage (TC) is of extreme importance because the target has to be eradicated with "one shot", and "quiet respiration" cannot be guaranteed. For this reason, we did not adopt the PTV specified by Blomgen and Lax et al. [1,2]. Another method for solving the problem of respiratory motion, administering high-frequency jet ventilation (HFJV) under general anesthesia, has proven effective for the total immobilization of targets in the liver [19] and is now being tested for SBRT of lung tumors.

Local irradiation alone for stage I NSCLC is a controversial issue. Several publications describe only a low risk for regional lymph-node relapse after careful staging [20-23]. In the light of the average age and the health conditions of our patient collective, we decided to dispense with mediastinal irradiation. No regional lymph-node relapses have been observed thus far.

Biologically equivalent doses (BED) can be calculated with the use of linear quadratic modeling, but this technique itself was derived from experiments with fractionation that were conducted in vitro or in vivo. In a recent article, Fowler et al. [24] attempted to develop a radiobiological model for hypofractionated high, single doses like those used in stereotactic body radiation therapy. But Fowler also clearly states that "linear quadratic (LQ) modelling is employed with only the standard assumption ... that LQ-modelling is valid up to 23 Gy per fraction." Fowler concedes that "ongoing clinical trials from an increasing number of centers will be reporting the results of tumor control and complications from this new modality of biologically higher doses." These statements from an authoritative radiobiologist demonstrate that this is new ground which has yet to be empirically evaluated. For this reason, we prefer to refrain from speculating on the radiobiological efficacy of our single-dose irradiation in comparison to hypofractionated stereotaxis schemes.

## Conclusion

Stereotactic single-dose irradiation could become an effective, non-invasive alternative to conventional surgery on metastases in the lungs. This alternative treatment involves few side effects but about 90% local control rates. Possible further indications might include the eradication of peripheral stage I non-small cell lung cancer. We have demonstrated the efficacy, feasibility, and safety of this approach in medically inoperable patients. Further exploration of the approach is warranted.

## Competing interests

The author(s) declare that they have no competing interests.

## Authors' contributions

PF and H-J K: were responsible for conception, design and clinical treatment.

WM: performed the statistical analysis and was clinically responsible for treatment planning and dose calculation.

UH and WD: were the main diagnostic radiologists who analyzed the CT scans during follow-up.

W E-R, AC and ES: cared for the patients as lung specialists and were responsible for measuring the respiratory functions during follow-up.
